# Evaluation of eight formulas for LDL-C estimation in Iranian subjects with different metabolic health statuses

**DOI:** 10.1186/s12944-019-1178-1

**Published:** 2019-12-28

**Authors:** Azam Karkhaneh, Molood Bagherieh, Solmaz Sadeghi, Asma Kheirollahi

**Affiliations:** 1 Reference Laboratory, Social Security Organization, Tehran, Iran; 2 Department of Clinical Biochemistry, School of Medicine, Tehran University of Medical Sciences, Tehran, Iran; 3 Students’ Scientific Research Center, Tehran University of Medical Sciences, Tehran, Iran; 4 Department of Medical Biotechnology, School of Advanced Technologies in Medicine, Tehran University of Medical Sciences, Tehran, Iran; 5 Department of Comparative Biosciences, Faculty of Veterinary Medicine, University of Tehran, Tehran, Iran

**Keywords:** LDL-cholesterol, Hattori formula, de Cordova formula, Freidewald formula

## Abstract

**Background:**

Considering the crucial role of low-density lipoprotein-cholesterol (LDL-C) concentration in determining cardiovascular risk, the accuracy of LDL-C estimation is essential. To date, various types of formulae have been introduced, albeit their accuracy has not been assessed in varied populations. In this study, the accuracy of eight formulae for LDL-C estimation was evaluated in an Iranian population.

**Methods:**

A data set of 2752 individuals was included in the study and all samples were analyzed in term of lipid profiles using direct homogeneous assay. The population was divided into various subgroups based on the triglyceride (TG), high-density lipoprotein- cholesterol (HDL-C), total cholesterol (TC), fasting blood sugar (FBS) and age values and estimated LDL-C values by Friedewald, Chen, de Cordova, Vujovic, Anandaraja, Hattori, Ahmadi, and Puavillai equations were compared to the directly measured LDL-C in each subgroup.

**Results:**

Estimated LDL-C values by Puavillai formulae showed an insignificant difference compared to the directly measured LDL-C in subjects with high level of TG. However, for TG range < 3.38 mmol/L and high levels of HDL-C, the difference between the means of estimated LDL-C by Hattori and de Cordova formulas, and directly measured LDL-C was relatively lower than other equations. In addition, estimated LDL-C by Hattori and de Cordova formulae had insignificant differences as compared to the direct LDL-C at some levels of cholesterol, the normal level of FBS and some age ranges.

**Conclusions:**

Therefore, it seems that Hattori and de Cordova formulas can be considered as the best alternatives for LDL-C direct measurement in the Iranian population, especially for healthy subjects.

## Introduction

Low-density lipoprotein (LDL) is a particle consists of outer phospholipids, apolipoproteins, free cholesterol and inner triglycerides (TGs) and cholesterol ester, which carries cholesterol from the liver to the peripheral tissues [1]. Coronary heart disease (CHD) is the leading cause of death worldwide [2]. The National Cholesterol Education Programme’s (NCEP) Adult Treatment Panel III (ATP III) has advised the LDL-cholesterol (LDL-C) as the major laboratory parameter for cardiovascular risk assessment and a main therapeutic target [3]. Hence, there is a need for a proper assessment of the serum levels of LDL-C.

The reference method for measurement of LDL-C, ultracentrifugation-polyanion precipitation / Beta Quantification (ßQ), is not suitable for routine laboratory testing because it is an expensive, laborious, tough and time-consuming technique, also requires large sample volumes [4]. Over the past decades, direct homogeneous assays have been developed for measurement of LDL-C concentration with a satisfactory degree of accuracy and precision as compared to the reference method [5]. However, mainly due to economic reasons, instead of the direct measurement of LDL-C, the calculation methods are widely used in clinical laboratories particularly in developing countries. More than 40 years ago, Friedewald Formula was developed based on fasting serum measurements of triglycerides (TG), high-density lipoprotein cholesterol (HDL-C) and total cholesterol (TC) and until now is the most commonly used method to estimate LDL-C in medical laboratories [6]. Some conditions such as TG >4.51 mmol/L and disorders related to lipoproteins decrease the accuracy of the Friedewald equation in the LDL-C estimation [6]. In addition to Friedewald Formula, there are several other formulas for calculation of LDL-C such as Chen, de Cordova, Vujovic, Anandaraja, Hattori, Ahmadi, and Puavillai, which have not been validated in varied populations [7–13].

The aim of this study was to compare the LDL-C calculated by Friedewald, Chen, de Cordova, Vujovic, Anandaraja, Hattori, Ahmadi and Puavillai formulas with the measured LDL-C by direct homogeneous assay over a wide range of TG, TC, FBS, HDL-C and age levels in Iranian subjects with the assumption that the results obtained by direct assays are the most accurate. We used a large sample size to validate the application of eight equations to calculate LDL-C in our population of outpatients. The results of this investigation showed that Hattori and de Cordova formulas have the best performance in the Iranian population.

## Materials and methods

### Study population

A data set of 2752 individuals (consists of 1915 females and 837 males, aged 4–92 years) from Tehran city was included in the study. This study was approved by the Ethics Committee of Tehran University of Medical Sciences (IR.TUMS.VCR.REC.1398.374). Blood samples were obtained at the Reference Laboratory of Social Security Organization in Tehran city (Iran). The laboratory participates in the external quality assessment with PISHGAM IRANIAN EQAS.

### Lipid profile analysis

The blood was collected in plain tubes after a 12-h fast and then centrifuged at 3000 rpm for 15 min. All samples were analyzed in term of lipid profiles comprising HDL-C, LDL-C, TG and TC. In addition, FBS values of all subjects –with some exception- were measured. Lipid profiles were determined by the standard homogenous enzymatic method using an automatic chemistry analyzer (Hitachi 902, Roche®) and the calibrating and internal controls were provided by the Pars Azmon Company.

TC was measured enzymatically in a series of coupled reactions by cholesteryl ester hydrolase, cholesterol oxidase and peroxidase, respectively. Produced H2O2 in the second reaction was measured quantitatively by a peroxidase catalyzed reaction that produces a color. Finally, absorbance was measured at 500 nm [14]. For TG measurement, triglycerides were hydrolyzed to produce glycerol using a series of coupled reactions. Then, glycerol was oxidized using glycerol oxidase and produced H2O2 was measured as described above for cholesterol [14]. HDL-C was measured in serum by the direct method. To remove apo B from the assay, the apoB containing lipoproteins in the specimen were treated with a blocking reagent. Sulfated alpha-cyclodextrin, polyethylene glycol-coupled cholesteryl esterase and cholesterol oxidase were used for the HDL-C measurement [14]. In the direct LDL-C measurement, firstly LDL was separated from the chylomicrons, VLDL, and HDL-C and then LDL-C was measured using an enzymatic colorimetric assay.

The population was divided into various subgroups based on the TG, HDL-C, TC, FBS and age values. There were five levels of TG (<0.56, 0.56–1.69, 1.70–3.38, 3.39–4.51 and > 4.51 mmol/L), three levels of HDL-C (<1.03, 1.03–1.52, ≥1.55 mmol/L), three levels of TC (<5.17, 5.17–6.18, ≥6.20 mmol/L), three levels of FBS (<5.49, 5.55–6.93 and ≥ 6.99 mmol/L), and four strata based on age (<20, 20–39, 40–59 and ≥ 60 years). For each subject, the LDL-C was calculated using all the following eight formulas:

Friedewald: LDL-C = TC – HDL-C – 0.2 × TG [6] Chen: LDL-C = (TC – HDL-C) × 0.9 – (TG × 0.1) [10]

de Cordova: LDL-C = 0.7516 × (TC – HDL-C) [12].

Vujovic: LDL-C = TC – HDL-C – (TG/6.85) [11].

Anandaraja: LDL-C = (0.9 × TC) – (0.9 × TG/5) – 28 [8].

Hattori: LDL-C = (0.94 × TC) – (0.94 × HDL-C) – (0.19 × TG) [7].

Ahmadi: LDL-C = TC/1.19 + TG/1.9-HDL-C/1.1 [9]

Puavillai: LDL-C = TC–HDL-C–TG/6 [13].

Finally, the directly measured LDL-C and the estimated-LDL-C by various formulas were compared in all strata.

### Statistical analysis

Statistical analysis was performed using SPSS Statistics 16.0. Continuous data were expressed as a mean and standard deviation. Paired Student’s t-test was used to compare the difference between LDL-C concentration measured by the direct method and various formulas. The level of significance was taken as *P* < 0.05 and the correlation was calculated using Pearson’s correlation test.

## Results

**Fig. 1:**
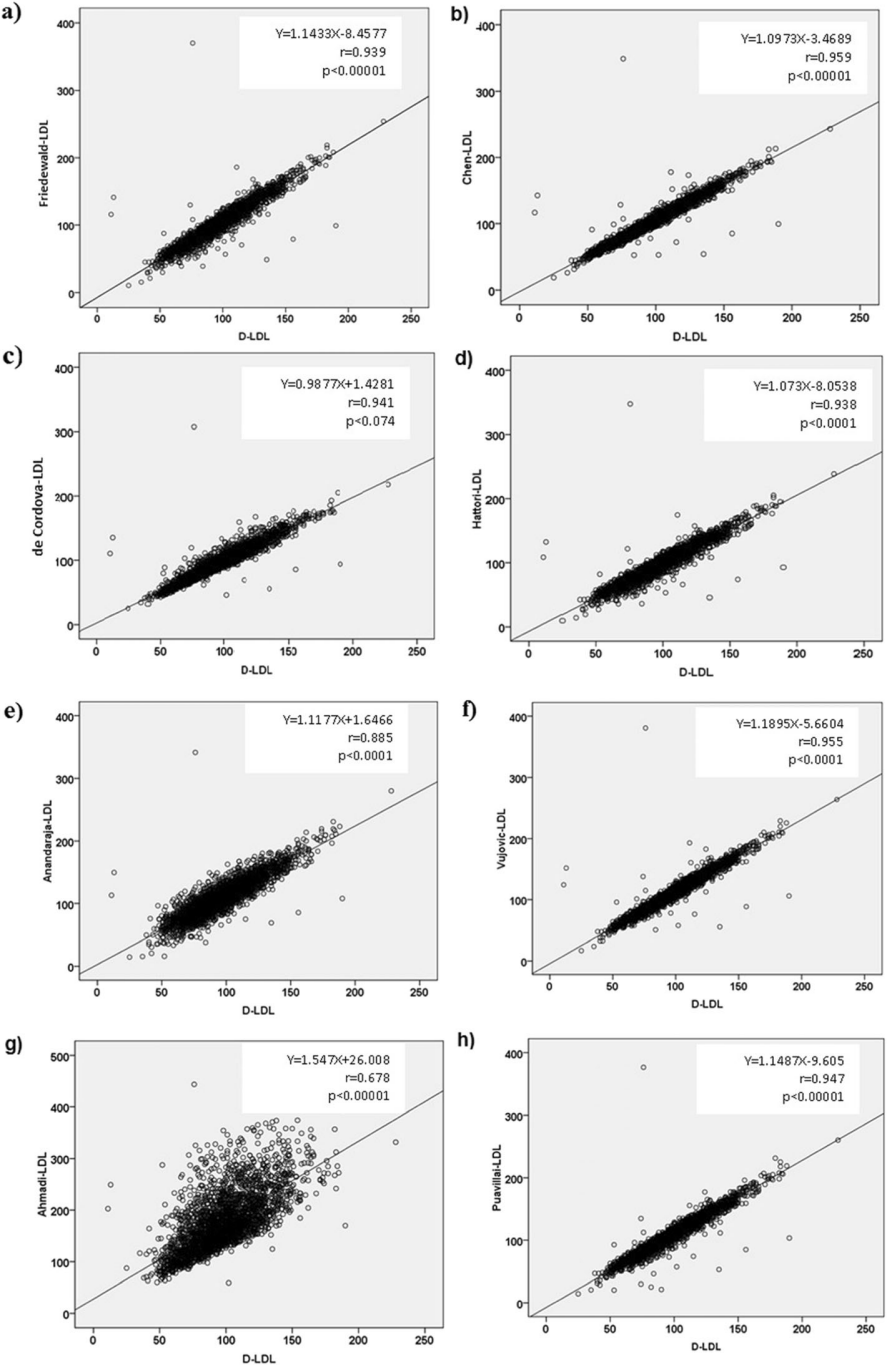
Correlation of calculated LDL by Friedewald (**a**), Chen (**b**), de Cordova (**c**), Hattori (**d**), Anandaraja (**e**), Vujovic (**f**), Ahmadi (**g**) and Puavillai (**h**) formulas with directly measured LDL

Demographic details of the participants are shown in Table 1. In the presented study, total 2752 lipid profiles were taken, out of this %69.6 were females and %30.4 were males. The mean age of participants was calculated as 49.9 ± 17 years. Due to its large size, our database involves healthy individuals, hyperlipidemic subjects, diabetics and people with other metabolic disorders. As shown in Fig. 1, calculated LDL-C concentrations by all equations -with the exception of Ahmadi formula- showed good correlation with the direct LDL-C. However, the mean value of calculated LDL-C by all equations had a significant difference with directly measured LDL-C (Table 1).

**Table 1:** Demographic details of the study subjects

Parameter	Mean ± SD (mmol/L)	Mean difference (mmol/L)	Correlation (r)	*p* value
Age	49.9 ± 17 (year)			
FBS	6.49 ± 2.52			
TG	1.64 ± 1.20			
TC	4.72 ± 0.99			
HDL-C	1.31 ± 0.28			
Direct LDL-C	2.53 ± 0.63			
Friedewald LDL-C	2.66 ± 0.79	0.13	0.927	0.00001
Chen LDL-C	2.69 ± 0.73	0.16	0.955	0.00001
de Cordova LDL-C	2.56 ± 0.70	0.03	0.902	0.00001
Hattori LDL-C	2.49 ± 0.74	−0.03	0.954	0.00001
Anandaraja LDL-C	2.85 ± 0.82	0.32	0.873	0.00001
Vujovic LDL-C	2.86 ± 0.79	0.33	0.926	0.00001
Ahmadi LDL-C	4.76 ± 1.89	2.22	0.519	0.00001
Puavillai LDL-C	2.78 ± 0.79	0.25	0.947	0.00001

### Estimation of LDL-C in five subgroups based on TG ranges

All lipid profiles were grouped into five TG ranges i.e., <0.56, 0.56–1.69, 1.70–3.38, 3.39–4.51 and > 4.51 mmol/L. As shown in Table 2, Friedewald formula underestimates and overestimates LDL-C at TG > 3.38 mmol/L levels and TG < 3.38 mmol/L levels, respectively. Also, Chen and Vujovic formulas overestimated LDL-C at all TG levels and de Cordova formula showed underestimation for TG < 150 mmol/L strata and overestimation for TG > 1.69 mmol/L levels. In contrast to the de Cordova formula, the Hattori formula overestimated LDL-C at TG < 1.69 mmol/L levels and underestimated LDL-C at TG > 1.69 mmol/L levels. Calculated LDL-C by Anandaraja formula showed overestimation at TG < 3.38 mmol/L levels and underestimation at TG > 3.38 mmol/L levels. Calculated LDL-C by Ahmadi formula indicated extreme overestimation with poor correlation. Although, the Puavillai formula showed an overestimation at TG < 3.38 mmol/L levels, estimated LDL-C by this equation in subjects with high level of TG (>3.38 mmol/L) had insignificant difference with the direct method.

**Table 2:** Mean (±SD) values of calculated LDL-C by eight formulae for various TG ranges and their correlation with directly measured LDL-C

Method	Mean ± SD (mmol/L)	Mean Difference (mmol/L)	Correlation (r)	*p*-value obtained by paired t-test
TG < 0.56 mmol/L (*n* = 66)				
Direct LDL-C	1.84 ± 0.43			
Friedewald LDL-C	2.05 ± 0.50	0.21	0.913	0.0001
Chen LDL-C	1.93 ± 0.46	0.09	0.908	0.0001
de Cordova LDL-C	1.71 ± 0.39	−0.13	0.902	0.0001
Hattori LDL-C	1.92 ± 0.47	0.08	0.913	0.001
Anandaraja LDL-C	2.45 ± 0.56	0.61	0.865	0.0001
Vujovic LDL-C	2.11 ± 0.50	0.27	0.910	0.0001
Ahmadi LDL-C	2.39 ± 0.47	0.55	0.857	0.0001
Puavillai LDL-C	2.09 ± 50	0.24	0.911	0.0001
TG 0.56–1.69 mmol/L (*n* = 1727)				
Direct LDL-C	2.43 ± 0.59			
Friedewald LDL-C	2.63 ± 0.72	0.20	0.967	0.0001
Chen LDL-C	2.57 ± 0.66	0.14	0.973	0.0001
de Cordova LDL-C	2.36 ± 0.57	−0.07	0.971	0.0001
Hattori LDL-C	2.47 ± 0.68	0.03	0.967	0.0001
Anandaraja LDL-C	2.88 ± 0.75	0.44	0.915	0.0001
Vujovic LDL-C	2.77 ± 0.73	0.33	0.971	0.0001
Ahmadi LDL-C	3.88 ± 0.84	1.45	0.852	0.0001
Puavillai LDL-C	2.7 ± 0.72	0.28	0.971	0.0001
TG 1.7–3.38 mmol/L (*n* = 817)				
Direct LDL-C	2.75 ± 0.66			
Friedewald LDL-C	2.81 ± 0.85	0.06	0.898	0.0001
Chen LDL-C	2.95 ± 0.77	0.19	0.903	0.0001
de Cordova LDL-C	2.89 ± 0.66	0.14	0.897	0.0001
Hattori LDL-C	2.63 ± 0.80	−0.11	0.897	0.0001
Anandaraja LDL-C	2.90 ± 0.90	0.15	0.894	0.0001
Vujovic LDL-C	3.09 ± 0.85	0.34	0.902	0.0001
Ahmadi LDL-C	5.89 ± 1.00	3.14	0.712	0.0001
Puavillai LDL-C	2.99 ± 0.85	0.23	0.922	0.0001
TG 3.39–4.51 mmol/L (*n* = 77)				
Direct LDL-C	2.79 ± 0.68			
Friedewald LDL-C	2.57 ± 0.92	−0.21	0.976	0.0001
Chen LDL-C	3.02 ± 0.81	0.23	0.978	0.0001
de Cordova LDL-C	3.27 ± 0.67	0.48	0.973	0.0001
Hattori LDL-C	2.40 ± 0.86	−0.39	0.976	0.0001
Anandaraja LDL-C	2.60 ± 0.98	−0.18	0.978	0.0001
Vujovic LDL-C	3.05 ± 0.91	0.26	0.978	0.0001
Ahmadi LDL-C	8.27 ± 0.80	5.48	0.793	0.0001
Puavillai LDL-C	2.87 ± 0.92	0.07	0.977	0.020
TG > 4.51 mmol/L (*n* = 65)				
Direct LDL-C	2.59 ± 0.63			0.0001
Friedewald LDL-C	2.07 ± 1.06	−0.52	0.884	0.0001
Chen LDL-C	3.04 ± 0.83	0.44	0.886	0.0001
de Cordova LDL-C	3.77 ± 0.80	1.17	0.622	0.0001
Hattori LDL-C	1.92 ± 1.01	−0.67	0.882	0.0001
Anandaraja LDL-C	2.11 ± 1.13	−0.48	0.913	0.0001
Vujovic LDL-C	2.87 ± 0.95	0.27	0.909	0.0001
Ahmadi LDL-C	11.88 ± 3.3	9.28	−0.053	0.0001
Puavillai LDL-C	2.56 ± 0.99	−0.03	0.906	0.592

### Estimation of LDL-C in three subgroups based on HDL-C ranges

The population was divided into three HDL-C ranges (<1.03, 1.03–1.52, ≥1.55 mmol/L) and the values of calculated LDL-C by formulas were compared to the directly measured LDL-C in each subgroup (Table 3). According to the results, Friedewald and Anandaraja equations showed underestimation and overestimation in subjects with HDL-C < 1.03 mmol/L and ≥ 1.03 mmol/L, respectively. Hattori equation underestimated and overestimated LDL-C in subjects with HDL-C < 1.55 mmol/L and ≥ 1.55 mmol/L, respectively. In contrast, the results of de Cordova formula indicated overestimation and underestimation for HDL-C < 1.55 mmol/L and ≥ 1.55 mmol/L levels, respectively. Estimated LDL-C by Chen, Puavillai, Vujovic and especially Ahmadi formulas was higher than the direct LDL-C without considering the state of HDL-C.

**Table 3:** Mean (±SD) values of calculated LDL-C by eight formulae for various HDL-C ranges and their correlation with directly measured LDL-C

Method	Mean ± SD (mmol/L)	Mean Difference (mmol/L)	Correlation (r)	*p*-value obtained by paired t-test
HDL-C < 1.03 mmol/L (*n* = 382)				
Direct LDL-C	2.22 ± 0.47			
Friedewald LDL-C	2.16 ± 0.67	−0.05	0.902	0.007
Chen LDL-C	2.38 ± 0.58	0.16	0.911	0.0001
de Cordova LDL-C	2.44 ± 0.65	0.22	0.675	0.0001
Hattori LDL-C	2.02 ± 0.63	−0.19	0.899	0.0001
Anandaraja LDL-C	2.04 ± 0.64	−0.17	0.901	0.0001
Vujovic LDL-C	2.45 ± 0.64	0.23	0.940	0.0001
Ahmadi LDL-C	5.55 ± 0.67	3.33	0.160	0.0001
Puavillai LDL-C	2.34 ± 0.64	0.12	0.933	0.0001
HDL-C 1.03–1.52 mmol/L (*n* = 1837)				
Direct LDL-C	2.54 ± 0.62			
Friedewald LDL-C	2.68 ± 0.74	0.13	0.904	0.0001
Chen LDL-C	2.70 ± 0.71	0.16	0.933	0.0001
de Cordova LDL-C	2.56 ± 0.68	0.02	0.905	0.0001
Hattori LDL-C	2.51 ± 0.70	−0.02	0.903	0.0001
Anandaraja LDL-C	2.83 ± 0.70	0.29	0.886	0.0001
Vujovic LDL-C	2.88 ± 0.77	0.33	0.926	0.0001
Ahmadi LDL-C	4.72 ± 1.72	2.17	0.631	0.0001
Puavillai LDL-C	2.80 ± 0.76	0.26	0.933	0.0001
HDL-C ≥ 1.55 mmol/L (*n* = 533)				
Direct LDL-C	2.70 ± 0.73			
Friedewald LDL-C	2.93 ± 0.85	0.23	0.974	0.0001
Chen LDL-C	2.87 ± 0.82	0.16	0.983	0.0001
de Cordova LDL-C	2.63 ± 0.76	−0.06	0.971	0.0001
Hattori LDL-C	2.75 ± 0.80	0.05	0.973	0.0001
Anandaraja LDL-C	3.48 ± 0.79	0.77	0.947	0.0001
Vujovic LDL-C	3.091 ± 0.89	0.38	0.982	0.0001
Ahmadi LDL-C	4.32 ± 1.55	1.62	0.806	0.0001
Puavillai LDL-C	3.03 ± 0.87	0.32	0.980	0.0001

### Estimation of LDL-C in three subgroups based on TC ranges

Another subgroup was created based on the TC values. In this regard, all data were divided into three levels (< 5.17, 5.17–6.18, ≥6.20 mmol/L), which are presented in Table 4. As it can be deduced from the results, estimated LDL-C values by Hattori formula indicated negligible differences compared to the direct method at high levels of TC (≥5.17 mmol/L). Although de Cordova equation had an insignificant difference with the direct method in normal ranges of TC (<5.17 mmol/L) (*P* = 0.354), it showed a positive bias in subjects with higher levels of TC. All other formulas overestimated LDL-C values in all three levels of TC.

**Table 4:** Mean (±SD) values of calculated LDL-C by eight formulae for various TC ranges and their correlation with directly measured LDL-C

Method	Mean ± SD (mmol/L)	Mean Difference (mmol/L)	Correlation (r)	p-value obtained by paired t-test
TC < 5.17 mmol/L (*n* = 1903)				
Direct LDL-C	2.22 ± 0.42			
Friedewald LDL-C	2.30 ± 0.52	0.08	0.885	0.0001
Chen LDL-C	2.33 ± 0.47	0.11	0.932	0.0001
de Cordova LDL-C	2.21 ± 0.43	−0.01	0.886	0.354
Hattori LDL-C	2.15 ± 0.49	−0.06	0.883	0.0001
Anandaraja LDL-C	2.48 ± 0.55	0.26	0.760	0.0001
Vujovic LDL-C	2.21 ± 0.52	−0.01	0.923	0.0001
Ahmadi LDL-C	4.08 ± 1.21	1.86	0.446	0.0001
Puavillai LDL-C	2.41 ± 0.52	0.19	0.920	0.0001
TC 5.17–6.18 mmol/L (*n* = 634)				
Direct LDL-C	3.05 ± 0.31			
Friedewald LDL-C	3.27 ± 0.47	0.21	0.771	0.0001
Chen LDL-C	3.30 ± 0.30	0.24	0.792	0.0001
de Cordova LDL-C	3.13 ± 0.29	0.07	0.400	0.0001
Hattori LDL-C	3.06 ± 0.44	0.01	0.769	0.327
Anandaraja LDL-C	3.48 ± 0.51	0.42	0.584	0.0001
Vujovic LDL-C	3.51 ± 0.37	0.45	0.809	0.0001
Ahmadi LDL-C	3.17 ± 1.65	0.12	−0.249	0.0001
Puavillai LDL-C	3.41 ± 0.40	0.36	0.879	0.0001
TC ≥ 6.20 mmol/L (*n* = 215)				
Direct LDL-C	3.72 ± 0.52			
Friedewald LDL-C	4.01 ± 0.91	0.29	0.704	0.0001
Chen LDL-C	4.13 ± 0.61	0.41	0.602	0.0001
de Cordova LDL-C	3.99 ± 0.54	0.27	0.161	0.0001
Hattori LDL-C	3.76 ± 0.87	0.04	0.705	0.337
Anandaraja LDL-C	4.24 ± 0.94	0.51	0.719	0.0001
Vujovic LDL-C	4.36 ± 0.75	0.64	0.668	0.0001
Ahmadi LDL-C	7.77 ± 2.92	4.05	−0.445	0.0001
Puavillai LDL-C	4.23 ± 0.81	0.51	0.689	0.0001

### Estimation of LDL-C in three subgroups based on FBS ranges

According to the FBS levels (mmol/L), the study subjects were divided into three groups including healthy (non-diabetic), pre-diabetic and diabetic individuals with the FBS level of <5.49, 5.55–6.93 and ≥ 6.99 mmol/L, respectively. Calculated LDL-C by various formulas was compared with the directly measured LDL-C at the different defined groups. Based on the results tabulated in Table 5, for non-diabetic subjects, the least mean differences compared to the direct method was observed in the estimated LDL-C values calculated by Hattori and de Cordova formulas with the mean differences of 0.01 mmol/L. *P*-value computation showed that the mean differences were statistically insignificant (*P* = 0.242 and *P* = 0.461, respectively). Moreover, the Freidewald equation showed the least mean differences compared to the direct method in diabetic subjects (mean difference = 0.95 and *P* = 0.043). While almost all formulas moderately overestimated LDL-C, the values obtained by the Ahmadi equation indicated highly overestimation with poor correlation in three levels of FBS.

**Table 5:** Mean (±SD) values of calculated LDL-C by eight formulae for three FBS levels and their correlation with directly measured LDL-C

Method	Mean ± SD (mmol/L)	Mean Difference (mmol/L)	Correlation (r)	p-value obtained by paired t-test
Non-diabetic: FBS < 5.49 mmol/L (*n* = 1505)				
Direct LDL-C	2.49 ± 0.62			
Friedewald LDL-C	2.67 ± 0.77	0.17	0.918	0.00001
Chen LDL-C	2.67 ± 0.72	0.17	0.942	0.00001
de Cordova LDL-C	2.50 ± 0.68	0.01	0.902	0.461
Hattori LDL-C	2.50 ± 0.72	0.01	0.917	0.242
Anandaraja LDL-C	2.88 ± 0.80	0.38	0.860	0.00001
Vujovic LDL-C	2.85 ± 0/78	0.35	0.940	0.00001
Ahmadi LDL-C	4.43 ± 1.71	1.94	0.563	0.00001
Puavillai LDL-C	2.78 ± 0.78	0.28	0.935	0.00001
Pre-diabetic: FBS 5.55–6.93 mmol/L (*n* = 543)				
Direct LDL-C	2.60 ± 0.62			
Friedewald LDL-C	2.72 ± 0.78	0.12	0.936	0.00001
Chen LDL-C	2.77 ± 0.71	0.17	0.961	0.00001
de Cordova LDL-C	2.65 ± 0.66	0.04	0.893	0.00001
Hattori LDL-C	2.55 ± 0.73	−0.05	0.935	0.00001
Anandaraja LDL-C	2.89 ± 0.82	0.28	0.887	0.00001
Vujovic LDL-C	2.94 ± 0.77	0.33	0.961	0.00001
Ahmadi LDL-C	4.99 ± 1.84	2.38	0.437	0.00001
Puavillai LDL-C	2.86 ± 0.77	0.25	0.970	0.00001
Diabetic: FBS ≥ 6.99 mmol/L (*n* = 637)				
Direct LDL-C	2.54 ± 0.66			
Friedewald LDL-C	2.56 ± 0.82	0.02	0.937	0.043
Chen LDL-C	2.68 ± 0.77	0.14	0.964	0.00001
de Cordova LDL-C	2.63 ± 0.74	0.08	0.893	0.00001
Hattori LDL-C	2.40 ± 0.77	−0.13	0.936	0.00001
Anandaraja LDL-C	2.74 ± 0.87	0.20	0.896	0.00001
Vujovic LDL-C	2.82 ± 0.83	0.27	0.965	0.00001
Ahmadi LDL-C	5.31 ± 2.14	2.77	0.492	0.00001
Puavillai LDL-C	2.72 ± 0.82	0.18	0.958	0.026

### Estimation of LDL-C in four subgroups based on age ranges

A total of 2754 lipid profiles were grouped into four age ranges, including <20, 20–39, 40–59 and ≥ 60 years, and the mean value obtained from all formulas were compared with the direct method. As shown in Table 6, the Ahmadi formula had a poor correlation with the direct measurement, while other formulas showed an appropriate correlation in all age ranges. At age < 20 years, all formulas -with the exception of Hattori and de Cordova formulas- overestimated LDL-C and showed significant difference with LDL-C measured by the direct method. At age 20–39 years, the difference between directly measured LDL-C and estimated LDL-C by de Cordova formula was not significant (*P* = 0.075). Taken together, it seems that the Hattori and de Cordova formulas with least mean differences were successfully able to estimate LDL-C level in all ages comparable to the direct method.

**Table 6:** Mean (±SD) values of calculated LDL-C by eight formulae for various age ranges and their correlation with directly measured LDL-C

Method	Mean ± SD (mmol/L)	Mean Difference (mmol/L)	Correlation (r)	p-value obtained by paired t-test
Age < 20 year (*n* = 100)				
Direct LDL-C	2.03 ± 0.50			
Friedewald LDL-C	2.14 ± 0.57	0.11	0.959	0.0001
Chen LDL-C	2.13 ± 0.57	0.10	0.986	0.0001
de Cordova LDL-C	1.99 ± 0.55	−0.03	0.974	0.006
Hattori LDL-C	2.01 ± 0.53	− 0.02	0.957	0.105
Anandaraja LDL-C	2.35 ± 0.59	0.31	0.862	0.0001
Vujovic LDL-C	2.28 ± 0.60	0.24	0.981	0.0001
Ahmadi LDL-C	3.50 ± 1.31	1.47	0.796	0.0001
Puavillai LDL-C	2.22 ± 0.59	0.19	0.974	0.0001
Age 20–39 year (*n* = 623)				
Direct LDL-C	2.37 ± 0.61			
Friedewald LDL-C	2.51 ± 0.70	0.13	0.940	0.0001
Chen LDL-C	2.53 ± 0.68	0.15	0.947	0.0001
de Cordova LDL-C	2.39 ± 0.66	0.01	0.922	0.075
Hattori LDL-C	2.35 ± 0.66	−0.01	0.938	0.043
Anandaraja LDL-C	2.72 ± 0.72	0.34	0.853	0.0001
Vujovic LDL-C	2.69 ± 0.72	0.32	0.974	0.0001
Ahmadi LDL-C	4.35 ± 1.87	1.97	0.584	0.00001
Puavillai LDL-C	2.62 ± 0.71	0.25	0.963	0.0001
Age between 40 to 59 year (*n* = 1138)				
Direct LDL-C	2.65 ± 0.61			
Friedewald LDL-C	2.78 ± 0.81	0.13	0.894	0.0001
Chen LDL-C	2.83 ± 0.73	0.18	0.921	0.0001
de Cordova LDL-C	2.70 ± 0.69	0.05	0.851	0.0001
Hattori LDL-C	2.61 ± 0.76	−0.03	0.892	0.0001
Anandaraja LDL-C	2.97 ± 0.85	0.32	0.858	0.0001
Vujovic LDL-C	3.00 ± 0.80	0.35	0.921	0.0001
Ahmadi LDL-C	5.05 ± 1.98	2.39	0.410	0.0001
Puavillai LDL-C	2.92 ± 0.80	0.27	0.914	0.0001
Age ≥ 60 year (*n* = 891)				
Direct LDL-C	2.54 ± 0.64			
Friedewald LDL-C	2.65 ± 0.80	0.11	0.957	0.0001
Chen LDL-C	2.69 ± 0.74	0.15	0.978	0.0001
de Cordova LDL-C	2.57 ± 0.68	0.03	0.931	0.0001
Hattori LDL-C	2.48 ± 0.75	−0.05	0.956	0.0001
Anandaraja LDL-C	2.85 ± 0.84	0.30	0.899	0.0001
Vujovic LDL-C	2.86 ± 0.80	0.31	0.977	0.0001
Ahmadi LDL-C	4.81 ± 1.72	2.26	0.546	0.0001
Puavillai LDL-C	2.78 ± 0.80	0.23	0.972	0.0001

## Discussion

Estimation of LDL-C is crucial in cardiovascular risk assessment for starting of dietary adjustments, drug intervention and monitoring [3]. Indeed, incorrectly determined LDL-C has adverse effect on CVD classification, therapy and outcomes in patients [3]. The reference method for determining LDL-C is time-consuming and not well suited to the clinical laboratory [4]. Therefore, precise LDL-C assessment is one of the most common challenges in the medical laboratory. The Friedewald equation is the most commonly used to estimate LDL-C and is relatively accurate for most people. However, it seems that this equation has inherent limitations, such as inaccurate LDL-C calculations in patients with hypertriglyceridemia, in those with very low levels of LDL-C (<2.4 mmol/L), in subjects with very low and/or high levels of TG (<1.12 and/or > 4.51 mmol/L), in patients with disorders related to lipoproteins (type III hyperlipoproteinemia), in patients with renal and liver failure, and in those with diabetes and other metabolic abnormalities [7, 10, 15–19]. Furthermore, several other equations have been introduced in an effort to address the drawbacks of the Friedewald equations. The underestimated LDL-C can lead to delay in initiation of appropriate lipid-lowering therapy in high-risk patients, whereas the overestimation of LDL-C will trigger unnecessary pharmacological therapy by placing the patient in higher risks strata. For this reason, finding an equation for the estimation of LDL-C in different populations with the best performance comparable to the direct measurement is of paramount importance.

To the best of our knowledge, this is the first attempt to evaluate the accuracy and reliability of various equations including Friedewald, Chen, de Cordova, Vujovic, Anandaraja, Ahmadi, Puavillai and Hattori formulas in Iranian subjects. The present study aims to answer the question of which of these formulas that are used to estimate LDL-C levels are more accurate compared to the direct measurement of LDL-C using a homogeneous assay. Our results showed the positive correlation between direct LDL-C and calculated LDL-C by almost all formulas (Fig. 1). This finding is in accordance with other studies, which report a correlation of calculated LDL-C using different formulas with LDL-C measured by different homogenous assays [11, 15–20]. However, the reliability of these formulas should be considered with respect to the levels of other biochemical parameters. In this context, we evaluated the correlation between actual LDL-C and calculated LDL-C by various equations at different levels of TG, FBS, HDL-C, TC and age. In this study, we included 2752 individuals (consists of 1915 females and 837 males, aged 4–92 years). Generally, women have greater HDL-C than age-matched men [21, 22], and puberty and menopause stages are associated with adverse alterations in plasma lipid levels due to changes in levels of sex hormones [23–26]. It should be noted that biological variations like sex and age could affect LDL-C, HDL-C and other lipid parameters in individual subjects and subsequently may alter the mean values in the population. Overall, plasma lipids are closely related to each other, so that gender and sex hormones alterations could change the level of all lipid parameters. As shown in Table 6, Hattori and de Cordova equations showed the best performance with the least mean differences at all age ranges. Thus, it seems that the performance of different methods is independent of age and sex hormonal alterations.

Most of these formulas at different levels of TG, FBS, TC, HDL-C, and age showed high correlation with the direct method, however, there might be a bias between estimated LDL-C and actual value of LDL-C. The results showed that Puavillai formula estimate LDL-C with high accuracy in subjects with high level of TG in the Iranian population. In this regard, Garule et al. concluded that the Puavillai formula is the most accurate formula and correlates with the direct method at all TG levels [27]. It was inferred from the results that the Friedewald-estimated LDL-C was 0.21 mmol/L higher than the directly measured LDL-C at TG levels of <0.56 mmol/L, while for TG > 4.51 mmol/L, mean value of the Freidewald-calculated LDL-C was approximately 0.52 mmol/L lower than the direct LDL-C. These results were consistent with the results previously reported by Mora et al., Martin et al., and Kannan et al. [17, 28, 29]. Although some studies have shown that the Friedewald equation performs better for certain groups of populations, the obtained results in this study indicated that this formula calculated LDL-C with a systematic overestimation over various levels of HDL-C, TC, FBS and age in the investigated Iranian population. However, after Hattori and deCordova formulas, the Friedewald formula showed the least mean difference with the direct method and outperformed other formulas for estimating LDL-C. In addition, according to the results of the Friedewald equation in diabetic subjects of this study, it could be suggested that this formula might be useful for LDL-C estimation in diabetic subjects. However, the use of the Friedewald equation in diabetic subjects has limited because of dyslipidemia in most of these patients.

The de Cordova and Hattori equations were the least accurate at low levels of HDL-C but at high HDL-C levels outperformed the other formulas. This finding was in accordance with obtained results by Martins et al. [19]. Hattori and deCordova formulas showed the lowest mean difference with the direct method and provide a significant advantage over other formulas in the Iranian population. However, these two formulas have to be used cautiously in hypertriglyceridemia and are not validated in subjects with TG > 3.39 mmol/L. It should be noted that the deCordova equation does not take into account TG levels, so it does not require fasting blood samples.

By considering the correlation and accuracy of different formulas, the Ahmadi equation showed the highest miscalculation with extreme overestimation in our population. This funding is contrary to the study performed by Ahmadi et.al, and it seems that this formula is not suitable to be used in the studied Iranian population [9]. The total mean difference of the estimated LDL-C by Chen, Vujovic, Anandaraja, and Puavillai equations was higher than the directly measured LDL-C and in most cases, this difference at pathologic levels of TG, HDL-C, TC, FBS, and older subjects was more than physiologic levels and younger subjects. Hence, the calculation of LDL-C by these formulas is not recommended, particularly for people over 40 years and those with an elevated level of TG, HDL-C, TC, and FBS.

The present study includes several inherent limitations. We had only access to the lipid profiles of the subjects, and clinical characteristics or clinical outcomes of patients in our sample were unknown. In addition, patients who received statin therapy and other cholesterol-lowering drugs were not excluded and there was missing information about renal, hepatic or cardiovascular diseases of subjects. In this study, estimated LDL-C by various formulas were not compared with the reference method i.e., ultracentrifuge and precipitation. Lipoprotein (a) level was not measured in blood samples, so, the effect of lipoprotein (a) had not been taken into account. Furthermore, we applied only one assay method for lipid profile measurement. Another limitation was the low number of samples for TG < 0.56 and TG > 4.51 mmol/L levels.

## Conclusions

Among the various formulas, the mean difference of estimated LDL-C by Hattori and de Cordova formulas with the directly measured LDL-C was noticeably lower than other formulas and it seems that these two equations have better performance than the others. However, it should be noted that in hypertriglyceridemia these two formulas fails to keep their supremacy, so they have to be used cautiously. Altogether, according to our results, Hattori and de Cordova formulas are the best alternatives for LDL-C direct measurement especially in young people with a normal range of TC and FBS.

## Data Availability

All data generated or analysed during this study are included in this published article.

## Abbreviations

Chol: Cholesterol
FBS: Fasting blood sugar
HDL-C: High-density lipoprotein-cholesterol
LDL-C: Low-density lipoprotein-cholesterol
TC: Total cholesterol
TG: Triglyceride
